# Adrenal involvement in the biostimulatory effect of bulls

**DOI:** 10.1186/1477-7827-5-33

**Published:** 2007-08-13

**Authors:** Shaun A Tauck, Jesse R Olsen, James G Berardinelli

**Affiliations:** 1 Department of Animal and Range Sciences, Montana State University, 119 Linfield Hall, Bozeman, MT, 59715, USA

## Abstract

**Background:**

The objective was to evaluate if cortisol concentrations are associated with the resumption of luteal activity in postpartum, primiparous cows exposed to bulls. The hypotheses were that 1) interval from start of exposure to resumption of luteal activity; 2) proportions of cows that resumed luteal function during the exposure period; and 3) cortisol concentrations do not differ among cows exposed or not exposed to bulls (Exp. 1), and cows continuously exposed to bull or steer urine (Exp. 2).

**Methods:**

In Exp. 1, 28 anovular cows were exposed (BE; n = 13) or not exposed (NE; n = 15) to bulls for 30 d at 58 d after calving. In Exp. 2, 38 anovular cows were fitted with a controlled urine delivery device at 45 d after calving and exposed continuously (24 h/d) to bull (BUE; n = 19) or steer (SUE; n = 19) urine. Length of exposure was ~64 d. Blood samples were collected from each cow on D 0 and every 3 d throughout exposure periods in both experiments and assayed for progesterone. Cortisol was assayed in samples collected on D 0, 8, 16, and 24 in Exp. 1; and, D 0, 19, 38, and 57 in Exp. 2.

**Results:**

In Exp. 1, interval from the start of exposure to resumption of luteal activity was shorter (P < 0.05) for BE cows than NE cows, similarly, more (P < 0.05) BE cows than NE cows resumed luteal function during the exposure period. In Exp. 2, there was no difference in intervals from the start of exposure to resumption of luteal activity and proportions of cows that resumed luteal function during the exposure period between BUE and SUE cows. In Exp. 1, there was no difference in cortisol concentrations between BE and NE cows at the start of the experiment (D 0), however, cortisol concentrations were greater (P < 0.05) in BE cows than NE cows on D 9, 18, and 27. In Exp. 2, cortisol concentrations were higher for BUE than SUE cows on D 0 (P < 0.05), thereafter cortisol decreased (P < 0.05) but did not differ between BUE and SUE cows.

**Conclusion:**

We conclude that the physical presence of bulls stimulates resumption of luteal activity and is coincident with increased cortisol concentrations, and hypothesize a possible association between adrenal activation and the biostimulatory effect of bulls.

## Background

Resumption of luteal function of primiparous, anovular suckled beef cows after calving is accelerated if cows are exposed to bulls [1] or excretory products of bulls [2]. The mechanism for this effect involves the hypothalamo-hypophyseal-ovarian (HPO) axis to increase LH pulse frequency [3] which in turn stimulates resumption of luteal function. Recently, research from our laboratory indicates that the mechanism for the biostimulatory effect of bulls is mediated by pheromones [2]. In mammals, male pheromones are predominantly carried in and released by urine [4]. However, the effects of pheromonal activation of the biostimulatory effect of bulls on neuroendocrine-endocrine events that precede resumption of luteal activity are not well understood.

Stress and stress hormones have long been associated with negative effects on reproductive activities. Stress associated release of cortisol and ACTH injections have been shown to cause a decrease in gonadotropin (LH and FSH) release in both cattle [5,6] and sheep [7]. However, Turner et al. [8] reported that repeated acute stress does not adversely affect estrous behavior, ovulation rates, and pregnancy rates in female pigs. In contrast there is an indication that the adrenal activation is involved in the response of female to male pheromones. Male urine sprayed into the nasal cavity of ovariectomized female rats stimulates an ACTH release causing the adrenal release of progesterone and corticosterone [9]. Furthermore, adrenalectomy attenuates the resumption of normal cycling activity in female mice exposed to male pheromone stimuli [9], indicating that adrenal activation may be involved in the physiological response of females to males pheromonal stimuli.

Two consecutive experiments were used to answer the question, "Is the biostimulatory effect of bulls associated with a rise in systemic cortisol concentrations?" The objective of this study was to determine if systemic cortisol concentrations are associated with the biostimulatory effect of bulls on resumption of luteal function in postpartum, anestrous beef cows. In Experiment 1 (Exp. 1) cows were exposed to either the continuous physical presence of bulls or not exposed to bulls. In Experiment 2 (Exp. 2) cows were continuously exposed to either bull urine or steer urine. We tested the hypotheses that: 1) interval from the start of exposure to resumption of luteal activity; 2) proportions of cows that resume luteal function during the exposure period; and 3) systemic cortisol concentrations do not differ between cows in Exp. 1 and between cows in Exp. 2.

## Methods

Two experiments were conducted at the Montana State University Livestock Teaching and Research Center, Bozeman. Animal care, handling, and protocols used in these experiments were approved by the Montana State University Institutional Large Animal Care and Use Committee. Experiments 1 and 2 were performed in 2003 and 2004, respectively.

### Animals

#### Experiment 1

Twenty-eight spring-calving two-yr-old Angus × Hereford primiparous suckled beef cows and four mature, epididymectomized Angus × Hereford bulls were used in this experiment. Cows and calves were maintained in a single pasture and had no contact with bulls or their excretory products during pregnancy and from calving until the start of the experiment. Average calving date for these cows was Feb. 16. Cows averaged 58 d postpartum at the start of the experiment (D -30), thirty days before the start of the breeding season (D 0). One week before the start of treatment cows were stratified by calving date, cow BW, calf birth weight, calf sex ratio, dystocia score, and BCS. Once cows were stratified they were assigned randomly to one of two treatments; exposure to mature bulls (BE; n = 13) or no bull exposure (NE; n = 15).

#### Experiment 2

Thirty-eight two-yr-old Angus × Hereford primiparous, suckled beef cows, four Angus × Hereford epididymectomized bulls, and four 1-yr-old Angus × Hereford steers were used in this experiment. Cows and calves had no contact with bulls or their excretory products from calving until the start of treatment. Average calving date was Feb. 9 and at the start of the experiment, March 21, cows averaged 40 d postpartum. Two weeks before treatment started cows were stratified by calving date, cow BW, calf birth weight, calf sex ratio, dystocia score, and BCS and fitted with a controlled urine delivery device (CUDD) [10]. Cows were then assigned randomly to either steer urine exposure (SUE; n = 19) or bull urine exposure (BUE; n = 19).

### Animal housing areas (Experiments 1 and 2)

At the start of each experiment cows were moved from a common pasture area into two lots, designated north and south by their geographic location. Each lot contained four pens (41 m × 18 m) that were similar in east-west configuration, bunk space, aspect, slope, and connection to open-shed shelters. Cows exposed to bulls (BE; Exp. 1) and cows exposed to bull urine (BUE; Exp.2) were housed in the north lot, cows not exposed to bulls (NE; Exp. 1) and cows exposed to steer urine (SUE; Exp. 2) were housed in the south lot. Cows were allowed to move between two pens within each lot. Lots were approximately 0.35 km apart. These lots and arrangements have proven to be effective in previous experiments involving bull-cow interactions [2].

In Exp. 1 bulls were housed with BE cows. In Exp. 2 bulls and steers were housed away from cows in two separate pens approximately 80 m apart and in a separate lot area north of the lots that housed cows by approximately 0.4 km.

### Bull exposure (Experiment 1)

Pens within the north lot were used for maintaining cows exposed to bulls (BE), while pens within the south lot were used for maintaining cows not exposed to bulls (NE). Cows assigned to either BE or NE treatments were placed into pens on D -30. Bull to cow ratio per pen was approximately 1:7.

### Bull and steer urine exposure (Experiment 2)

Pens within the north lot were used for maintaining cows exposed continuously to bull urine (BUE), while pens within the south lot were used for maintaining cows exposed continuously to steer urine (SUE). Continuous exposure of cows to bull and steer urine was accomplished with a CUDD. Details of the components and construction of CUDDs, and urine collection stanchions, urine collection devices, handling of urine from bulls and steers, and filling of CUDDs are given in Tauck et al. [10].

### Nutrition (Experiments 1 and 2)

Cows had free access to good quality, chopped mixed-grass alfalfa hay, and any pasture grasses that were available before the start of each experiment. Once cows and calves were moved into pens they were given free access to the same hay, 0.5 kg·hd^-1^·d^-1 ^cracked barley, water, and a trace mineral-salt supplement. The TDN of the diet exceeded the NRC requirement for lactating beef cows with a mature weight of 545 kg by approximately 18% [11]. In Experiment 1, bulls had access to the same ratio as cows. In Experiment 2, bulls had ad libitum access to fair quality, chopped barley hay. During collection periods, bulls were fed 0.5 kg of cracked barely and good quality, chopped mixed-grass alfalfa hay. Steers were fed a finishing ration that consisted of 70% concentrate and 30% roughage throughout the experiment.

### Blood sampling (Experiments 1 and 2)

To determine resumption of luteal activity blood samples were collected from each cow by jugular venepuncture at 3-d intervals from the start of the experiment to the start of the breeding season. Serum was assayed for progesterone concentration in duplicate using solid-phase RIA kits (Diagnostic Products Corp., Los Angeles, CA) validated for bovine serum in our laboratory [1]. Intra- and interassay CV for a serum pool that contained 0.42 ng/mL were < 10.0%, respectively; and for a pool that contained 3.1 ng/mL were < 7.0%, respectively, for blood samples from Exp. 1. Intra- and interassay CV for a serum pool that contained 2.6 ng/mL of progesterone were 0.4 and 7.4%, respectively; and 3.4 and 11.0%, respectively, for a pool that contained 7.5 ng/mL in Exp. 2. Progesterone concentration patterns were used to determine the occurrence of resumption of luteal activity and the intervals from the start of treatment to resumption of luteal cycling activity. An increase of progesterone concentration, above the average progesterone baseline of individual cows, in three consecutive samples that exceeded 1 ng/mL was used as the criteria to determine the occurrence of resumption of luteal activity. Intervals from the start of treatment to resumption of luteal activity were determined by the number of days from the treatment to the lowest inflection point before a rise in three consecutive samples that exceeded 1 ng/mL. Cows that failed to exhibit a rise in progesterone over three consecutive samples were assigned an interval from the start of treatment to the end of treatment.

Changes in overall cortisol concentration means were determined by blood samples collected in equally spaced intervals throughout either bull or bull urine exposure periods in Exp. 1 and 2. Blood samples obtained on Days 0, 8, 16, and 24 in Exp. 1 and Days 0, 19, 38, and 57 in Exp. 2 were assayed for cortisol concentrations using a solid-phase RIA kits (Diagnostic Products Corp., Los Angeles, CA, USA) validated for bovine serum in our laboratory [12]. Intra- and interassay CV for a serum pool that contained 0.08 ng/mL were 4.8 and 11%, respectively; and for a pool that contained 7.0 ng/mL were 2.0 and 5.4%, respectively.

### Statistical analyses

Calving date, cow BW, calf birth weight, calf sex ratio, dystocia score, and BCS were analyzed by separate ANOVA for a completely randomized design using PROC GLM of SAS (SAS Inst. Inc., Cary, NC, USA). The model included treatment and means were separated by the PDIFF procedure of SAS. Intervals from the start of treatment to resumption of luteal activity were analyzed by ANOVA for a completely randomized design using PROC GLM of SAS. Proportions of cows that resumed luteal activity by the end of the exposure period were analyzed by chi-square analyses using the PROC FREQ procedure of SAS.

Cortisol concentrations for each day of sampling of Experiments 1 and 2 were analyzed by separate ANOVA for each for a completely randomized split-plot design using PROC GLM of SAS. The main plot included treatment and animal within treatment. The animal within treatment variance component was used to test the effect of treatment. The sub-plot included day and the interaction of treatment and day. Means were separated by the PDIFF procedure of SAS.

## Results

### Experiment 1

Calving date, cow BW, calf sex ratio, and number of days postpartum did not differ (*P *> 0.10) among treatments. More (*P *< 0.05) cows exposed to the physical presence of bulls (100%) resumed luteal activity by the end of the experiment than cows not exposed to bulls (47% ; Table 1). The interval from the start of treatment to resumption of luteal activity was shorter (*P *< 0.05) for cows exposed to bulls (3.76 d) than cows not exposed to bulls (18.2 d).

**Table 1:** Characteristics, and luteal response of cows exposed or not exposed to bulls in Experiment 1.

	Treatments			
		
Variable	BE	NE	SEM^a^	*P *value
		
n	13	15		
Calving date^b^	54.69	50.27	15.88	0.47
Days postpartum at start of exposure	56.31	60.67	15.95	0.48
Cow BW (kg)	520.66	519.28	44.24	0.93
Cow BW change (kg)	-3.77	-12.91	14.03	0.10
Calf sex ratio^c^	0.62	0.73	0.48	0.52
Interval to resumption of luteal activity during the exposure period, d	3.76	18.2	11.56	< 0.01
% resuming luteal activity during the exposure period	100%	47%		0.01^d^

^a^SEM = Pooled standard error for means.

^b^Day of year.

^c^Calf Sex ratio = ratio of male to female calves, 1 = male and 0 = female.

^d^*X*^2 ^= 6.63, d. f. = 1.

**Figure 1:**
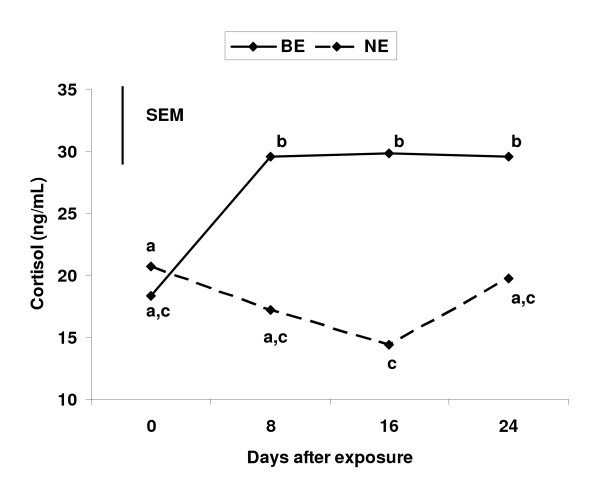
**Cortisol concentrations in 8-d intervals during the 30 d bull-exposure period in primiparous, suckled beef cows exposed (BE) or not exposed (NE) to bulls in Experiment 1**. Vertical line represent the pooled SEM (SEM = 8.3 ng/mL). Points that lack common letters differ (*P *< 0.05).

There was a treatment by day interaction (*P *< 0.01) for systemic cortisol concentrations. This was due to a rise in cortisol concentrations for cows exposed to the physical presence of bulls from D 0 to 9, while cows not exposed to bulls did not exhibit a rise in cortisol from D 0 to 9 (Figure 1). Thereafter, from D 9 to 24, cortisol concentrations in BE cows remained higher and more stable than cortisol concentrations in NE cows which decreased from D 0 to D 16 and increased from D 16 to D 24 (Figure 1).

### Experiment 2

Calving date, cow BW, calf birth weight, calf sex ratio, dystocia score, and BCS did not differ between cows exposed to bull urine (BUE) and steer urine (SUE) (Table 2). Similarly, cow BW and BCS change from the start of the exposure period until the end of the exposure period did not differ between BUE and SUE cows (Table 2). There was no difference in the intervals from the start of the exposure period to the resumption of luteal activity between BUE and SUE cows, 62.5 and 55.8 d respectively (Table 2). Likewise, proportions of cows that resumed luteal activity by the end of the exposure period did not differ between BUE and SUE cows, 15% and 33% respectively (Table 2).

**Table 2:** Characteristics, and luteal response of cows exposed to bull or steer urine in Experiment 2.

	Treatments			
		
Variable	BUE	SUE	SEM^a^	*P *value
		
n	19	19		
Calving date^b^	39	39	9.22	0.98
Cow BW (kg)	557	550	40.90	0.59
Cow BW change (kg)^c^	-37.20	-22.30	21.50	0.22
Calf BW (kg)	35	39	9.10	0.92
BCS	5.10	5.20	0.34	0.60
BCS change^c^	-0.04	-0.04	0.35	0.98
Calf sex ratio^d^	0.47	0.60	0.51	0.44
Dystocia score^e^	1.00	1.05	0.16	0.34
Interval to resumption of luteal activity during the exposure period, d	62.50	55.80	14.60	0.17
% resuming luteal activity during the exposure period	15%	33%		0.25^f^

^a^SEM = Pooled standard error for means.

^b^Day of year.

^c^Cow BW and BCS change are differences from the start of treatment to the end of treatment.

^d^Calf sex ratio = ratio of male to female calves, 1 = male and 0 = female.

^e^Dystocia Score: 0 = No assistance to 5 = Caesarean section.

^f^*X*^2 ^= 1.3, d.f. = 1. Table legend text.

**Figure 2:**
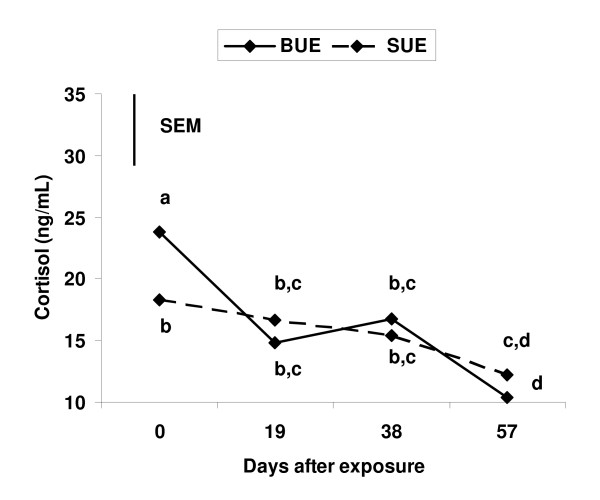
**Cortisol concentrations in 19-d intervals during the 57 d urine-exposure period in primiparous, suckled beef cows exposed to bull urine (BUE) or exposed to steer urine (SUE) in Experiment 2**. Vertical line represent the pooled SEM (SEM = 6.3 ng/mL). Points that lack common letters differ (*P *< 0.05).

There was a treatment by day interaction (*P *< 0.05) for patterns of cortisol concentrations. This interaction appeared to be caused by the difference in cortisol concentrations between BUE and SUE on D 0 and the larger decrease in cortisol between D 0 and 9 in BUE than in SUE cows (Figure 2). Nevertheless, cortisol concentrations in both BUE and SUE cows decreased (*P *< 0.05) during exposure period (Figure 2); and cortisol concentrations for any one day did not differ (*P *> 0.10) between BUE and SUE cows throughout the exposure period (Figure 2).

## Discussion

Postpartum, anovular cows exposed to excretory products of bulls for 12 h/d for 70 d mimicked the effect of the physical presence of bulls in accelerating resumption of ovulatory cycles [2]. This indicates that the biostimulatory effect of bulls involves a pheromonal mechanism. However, the physiological mechanism by which cows resume luteal function in response male pheromone stimuli is not well understood. The temporal release pattern of LH dictates resumption of luteal activity in postpartum anestrous cows; generally there is an increase pulse frequency and decrease in pulse amplitude of LH before anestrous cows resume ovarian cycling activity [13-15]. Fernandez et al. [3] reported that bull exposure accelerates the onset of LH release patterns that cause resumption of luteal activity in postpartum anestrous cows, consequently, cows exposed to bulls resume luteal activity earlier after calving than cows not exposed to bulls.

How does bull exposure stimulate and accelerate the onset of LH release patterns that cause resumption of luteal activity in postpartum, anovular, suckled cows? Studies in mice and rats indicate that adrenal activation is necessary for females to respond to male pheromone stimulation. Irregular cycling female mice and rats exposed to male urine exhibit a rise in gonadotropin (LH and FSH) secretion and return to regular ovarian cycling activity [9,16]. Marchlewska-Koj and Zacharczuck-Kakietek [17] reported circulating corticosterone concentrations of female mice rise within 10 min after exposure to male bedding. Furthermore, male urine sprayed into the nasal cavity of ovariectomized female rats stimulates an ACTH release causing the adrenal release of progesterone and corticosterone [9]. Adrenalectomized female mice do not respond to the effect of male urine on resumption of regular ovarian cycles [9]. Taken together these data indicate a close physiological association between the effect of male pheromones and adrenal activity.

To determine if systemic cortisol concentrations are associated with the biostimulatory effect of bulls we evaluated the effectiveness of either the physical presence of bulls or continuous bull urine exposure to stimulate resumption of luteal function in postpartum, anestrous beef cows. We found that cows in Exp. 1 resumed luteal function in response to the physical presence of bulls earlier than cows not exposed to bulls; however, in Exp. 2, neither the proportion of cows that resumed luteal function nor the interval to resumption of luteal activity differed between cows exposed to bull or steer urine. From these data we conclude that the physical presence of bulls caused a biostimulatory effect on resumption of luteal activity, whereas continuous bull urine exposure did not stimulate resumption of luteal function.

Cortisol concentrations in cows not exposed to bulls in Exp. 1 ranged from 21 to 16 ng/mL. These data are consistent with previous reports for systemic circulating cortisol concentration in cows 40 to 60 d postpartum, which ranged from 8 to 16 ng/mL [13,18,19]. However, we found that cows exposed to the physical presence of bulls in Exp. 1 exhibited a significant rise in systemic cortisol concentrations from 16 ng/mL on D 0, to 29 ng/mL throughout the remainder of the experiment. Wagner and Oxenreider [18] reported that cortisol is released in a diurnal pattern with a change of approximately ± 1 ng/mL every h and peak cortisol release occurring at 4:00 am and 4:00 pm. The diurnal pattern of cortisol release cannot explain the results observed in Exp. 1 because blood samples were collected within a 2 h period each sampling d between 9:00 am and 2:00 pm from cows in both treatments. It is possible that the increase in cortisol concentrations observed in Exp. 1 were due to an acute stress caused by the physical presence of bulls. Mean cortisol concentrations in cows that have been subjected to an acute physical stress, such as transportation, or cows that have been injected with adrenocorticotropic hormone (ACTH), to mimic a stress response, exhibit a rise in cortisol concentrations that meet or exceed 60 ng/mL [20,21]. Mean cortisol concentrations for cows in Exp. 1 and Exp. 2 were well below this concentration, indicating that cows used in these experiments did not exhibit a typical ACTH driven cortisol response and were not subjected to conditions that would cause a stress-like cortisol response.

In Exp. 2, we found that systemic cortisol concentrations decreased as time after calving increased for cows exposed to either bull or steer urine. This result is probably a photoperiod effect and is consistent with the results of Leining et al. [22] who reported that as photoperiod increased concentrations of circulating glucocorticoids decreased in bulls. Systemic cortisol concentrations did not differ between cows continuous exposed to mature bull urine or steer urine. This result might have been expected since the physical presence of bulls stimulated resumption of luteal activity in Exp. 1 while continuous bull urine exposure did not stimulate resumption of luteal function in Exp. 2. Taken together, these results provide compelling evidence that elevated cortisol concentrations in postpartum, anovular cows are a result of the biostimulatory effect of bulls. Thus, adrenal activation is a probable component of the pheromone-mediated biostimulatory effect of bulls for accelerating resumption of luteal function in postpartum, anovular beef cows.

## Conclusion

In conclusion, bull urine exposure had no effect on circulating cortisol concentration and did not reduce the postpartum anestrus interval from calving to the resumption of ovarian cycling activity or increase the proportion of cows cycling by the end of the exposure period. However, the physical presence of bulls elevated systemic cortisol concentrations and shortened the postpartum, anestrus interval to resumption of luteal function in postpartum, anovular cows. Thus, it is possible that cortisol is a critical marker and/or component of the bull pheromone biostimulatory pathway that stimulates resumption of luteal activity in postpartum, anestrous beef cows. This is the first study that indicates that adrenal activation may be involved with the biostimulatory effect of bulls. Further research is necessary to evaluate whether adrenal activation is a mediator or a consequence of the biostimulatory effect of bulls that initiates the neuroendocrine-endocrine cascade that accelerates resumption of ovulation and luteal function in postpartum, anestrous, suckled cows.

## Competing interests

The author(s) declare that they have no competing interests.

## Authors' contributions

SAT, JRO, and JGB took part in designing the experiments, performed the experiments, analyzing the data, and drafting the manuscript. All authors read and approved the final manuscript.
